# Impact of unplanned peritoneal dialysis start on patients' outcomes—A multicenter cohort study

**DOI:** 10.3389/fmed.2022.717385

**Published:** 2022-11-23

**Authors:** Kellen Thayanne Hangai, Roberto Pecoits-Filho, Peter G. Blake, Daniela Peruzzo da Silva, Pasqual Barretti, Thyago Proença de Moraes

**Affiliations:** ^1^Programa de Pós- Graduação em Ciências da Saúde-Pontifícia Universidade Católica do Paraná (PUCPR), Curitiba, Brazil; ^2^Division of Nephrology, University of Western Ontario, London, ON, Canada; ^3^Division of Nephrology, Department of Internal Medicine, Universidade Estadual Paulista (UNESP), Botucatu, Brazil

**Keywords:** unplanned peritoneal dialysis, urgent-start dialysis, early-start dialysis, peritoneal dialysis, BRAZPD

## Abstract

**Background:**

Patients with end-stage kidney disease (ESKD) who start unplanned dialysis therapy are more likely to be treated with hemodialysis (HD) using a central venous catheter, which has been associated with a greater risk of infections and other complications, as well as with a higher long-term risk of death. Urgent-start PD is an alternative that has been suggested as an option for starting dialysis in these cases, with potentially better patient outcomes. However, the definition of urgent-start PD is not homogeneous, and no study, to our knowledge, has compared clinical outcomes among urgent start, early start, and conventional start of PD. In this study, we aimed to compare these types of initiation of dialysis therapy in terms of a composite outcome of patient survival and technique failure.

**Methods:**

This is a retrospective, multicenter, cohort study, involving data from 122 PD clinics in Brazil. We used the following: Urgent-start groups refer to patients who initiated PD within 72 h after the PD catheter insertion; early-start groups are those starting PD from 72 h to 2 weeks after the catheter insertion; and conventional-start groups are those who used the PD catheter after 2 weeks from its insertion. We analyzed the composite endpoint of all causes of patient's mortality and technique failure (within the initial 90 days of PD therapy) using the following three different statistical models: multivariate Cox, Fine and Gay competing risk, and a multilevel model.

**Results:**

We included 509 patients with valid data across 68 PD clinics. There were 38 primary outcomes, comprising 25 deaths and 13 technique failures, with a total follow-up time of 1,393.3 months. Urgent-start PD had no association with the composite endpoint in all three models.

**Conclusion:**

Unplanned PD seems to be a safe and feasible option for treatment for patients with non-dialysis ESKD in urgent need of dialysis.

## Introduction

Chronic kidney disease (CKD) is a silent condition that frequently progresses to end-stage kidney failure (ESKD) without or with only minor symptoms. Consequently, many patients with ESKD are seen for the first time by a doctor in urgent conditions, such as electrolyte disturbances, hypervolemia, uremia, and other serious consequences of CKD. Of note, the 2020 United Renal Data System showed that one-third of patients with incident ESKD were reported to have received little or no pre-ESKD care (1). In addition, late referral, defined as the initiation of a kidney replacement therapy within 6 months of the first visit to the nephrologist, is common, with an incidence from 30 to 80% (2).

In urgent cases, unplanned dialysis start is the only option to save patients with ESKD. Individuals who start unplanned dialysis are more likely to be treated with hemodialysis (HD) using a central venous catheter (CVC) (3), which has been associated with a greater infection rate and other early complications, as well as a higher risk of long-term bad outcomes (4–6). Over the past decade, several authors have described their center's experiences with unplanned PD and the term “urgent-start” PD has emerged (7, 8). The studies comparing urgent-start PD with conventional-start HD have found equivalent results in terms of patient survival and even a lower risk of bacteremia (9–11). However, the definition of urgent-start PD was not standardized in the first reports, and only recently, it has taken shape and been established as the use of PD dialysis catheter up to 72 h after their insertion, while those between 3 and 14 days have been named as early-start PD (12).

No previous report, to our knowledge, has compared clinical outcomes among urgent-start, early-start, and conventional-start PD. The main objective of this study was to compare urgent-start PD with early-start PD and conventional-start PD in terms of a composite outcome of patient survival and technique failure, in a large multicenter cohort of PD patients.

## Materials and methods

This is a retrospective, multicenter, cohort study that included PD patients between November 2004 and January 2011 from 122 PD clinics in Brazil. This study was conducted in accordance with the Declaration of Helsinki. It was approved by the local ethics committee of the Pontificia Universidade Católica do Paraná (PUC-PR), register number 448. All patients signed an informed consent to participate. The aim of our study was to analyze whether the unplanned start of PD is associated with more adverse events, as defined by mortality and technique failure, the composite outcome of the study. The patients were divided into the following three groups: Group 1 (urgent-start), composed by patients who initiated PD within 72 h after the catheter insertion; Group 2 (early-start) with patients starting PD between 72 h and 2 weeks after the catheter insertion; and Group 3 (conventional-start) with patients initiating PD catheter use after 2 weeks of its insertion.

Data were obtained from the database of BRAZPD, a multicenter Brazilian cohort involving more than 7,000 incident PD patients, who were followed from November 2004 and January 2011 (13). Such data were reported monthly to a central computer using specific software designed for the study. We collected demographic and clinical data when the patients were included in the study (baseline). Laboratory and clinical data were also collected and reported every month except for residual renal function, which was only available at baseline. Data on the interval between catheter insertion and the first PD dialysis started to be recorded in 2008.

The eligibility criteria for this study were adult patients (>18 years old), those incident on PD (starting PD therapy at the same moment they were included in the study), and those with valid data on catheter insertion and its first use.

The variables captured and tested as covariates in the univariate analysis were as follows: age, gender, history of diabetes, hypertension, previous chronic hemodialysis, previous renal transplantation, peripheral artery disease, coronary artery disease, first PD modality, pre-dialysis care (defined as at least 3 months of treatment with a nephrologist before the initiation of dialysis), literacy (categorized into two groups with less than or with 4 or more years of formal study), residual renal function (defined as a daily urine volume >100 ml), body mass index, center experience (in patient-months), and the laboratory data as a continuous variable. Center experience was expressed in patient-months, i.e., the cumulative follow-up time of all patients from a center was obtained and the result divided by the number of years the center participating in the study.

Continuous variables were reported as mean ± standard deviation for normally distributed variables or percentage for categorical variables. Unpaired *Student's t-*test or *Chi-square* test was used for comparisons of groups' characteristics. The primary outcome was a composite endpoint of all-cause mortality and technique failure within the initial 90 days of therapy. In addition, both all-cause mortality and technique failure were analyzed separately using three different models: Cox regression, competing risk of Fine and Gray, and a three-level multilevel analysis. Patients alive at 3 months were treated as censored in all models, and those who dropped the study for any other causes different from the outcome of interest were treated as a competing event in the competing risk analysis. The three levels of the multilevel analysis are the patient (first level), the dialysis clinic (second level), and the city where the clinic is located (third level). The multivariable models were composed by the pre-defined groups, confounders with an alpha value < 0.10 in the univariate analysis and from traditional risk factors that did not match an alpha value < 0.10 in the univariate analysis. In the sequence, we performed a likelihood test to define which variables best fit the final model.

## Results

Considering the inclusion and exclusion criteria, our sample was composed of 509 patients. These individuals were distributed across 68 PD clinics. The characteristics of the study population were in general similar to the original BRAZPD cohort (13) except for the prevalence of patients with previous hemodialysis, which was less frequent in this work (Table 1).

**Table 1:** General baseline characteristics.

**Variable**	**BRAZPD cohort**	**Study population**	**Urgent-start**	**Early-start**	**Conventional-start**
	**(13) (*n* = 5,707)**	**(*n* = 509)**	**(*n* = 170)**	**(*n* = 153)**	**(*n* = 186)**
Age (years)	59 ± 16	61 ± 16	59 ± 17	64 ± 15	61 ± 15
Hypertension (yes)	73%	77%	80%	78%	75%
Center experience (pt-months)	42 ± 25^#^	46 ± 26^#^	43 ± 23^#^	43 ± 25^#^	52 ± 28
Coronary artery disease (yes)	21%	24%	24%	18%	28%
Diabetes (yes)	44%	46%	47%	45%	47%
Gender (Male)	48%	52%	56%	50%	51%
Literacy (< 4 years)	55%	64%	63%	65%	65%
Initial PD modality (APD)	46%	58%	59%	48%	64%
Residual renal function (yes)	65%	77,4%	80,6%	80,4%	72%
Peripheral artery disease (yes)	21%	15%	15%	10%	18%
Pre-dialysis care (yes)	51%	59%	48%	64%	65%
Previous hemodialysis (yes)	36%	6%^##^	4%^##^	4%^##^	11%^##^
Previous transplantation (yes)	2%	1%	1%	0%	1%
Race (White)	64%	71%	73%	76%	65%
BMI < 18.5 kg/m^2^	6%	3%	2%	3%	4%
>25 kg/m^2^	43%	46%	49%	49%	41%
Systolic BP (mmHg)	138 ± 24	136 ± 24	136 ± 24	137 ± 24	136 ± 23
Diastolic BP (mmHg)	83 ± 13	82 ± 14	82 ± 13	82 ± 14	82 ± 14
Hemoglobin(g/dL)	10.6 ± 2.0	10.5 ± 1.9	10.4 ± 2.1	10.5 ± 1.9	10.4 ± 1.8
Serum phosphate (mg/dl)	5.2 ± 1.6	5.2 ± 1.7	5.5 ± 1.7	5.0 ± 1.6	5.1 ± 1.7
Serum potassium (mEq/L)	4.7 ± 0.9	4.7 ± 0.9	4.8 ± 1.0	4.5 ± 0.8	4.7 ± 1.0

#*p* < 0.05 vs. Conventional, ##*p* < 0.01 vs. BRAZPD.

The mean age was 61 ± 16 years, 52% were male, and 46% of the patients were diabetics. The groups were composed as follows: urgent-start group with 170 patients, early-start group with 153 patients, and conventional-start group with 186 patients. The three groups were similar in most of the clinical and demographic characteristics. Two variables presented marked differences among groups: Center experience was lower for the urgent- and early-start groups compared with the conventional-start group and the prevalence of patients with pre-dialysis care was lower for the urgent-start group compared with the other two.

### Composite outcome

**Figure 1:**
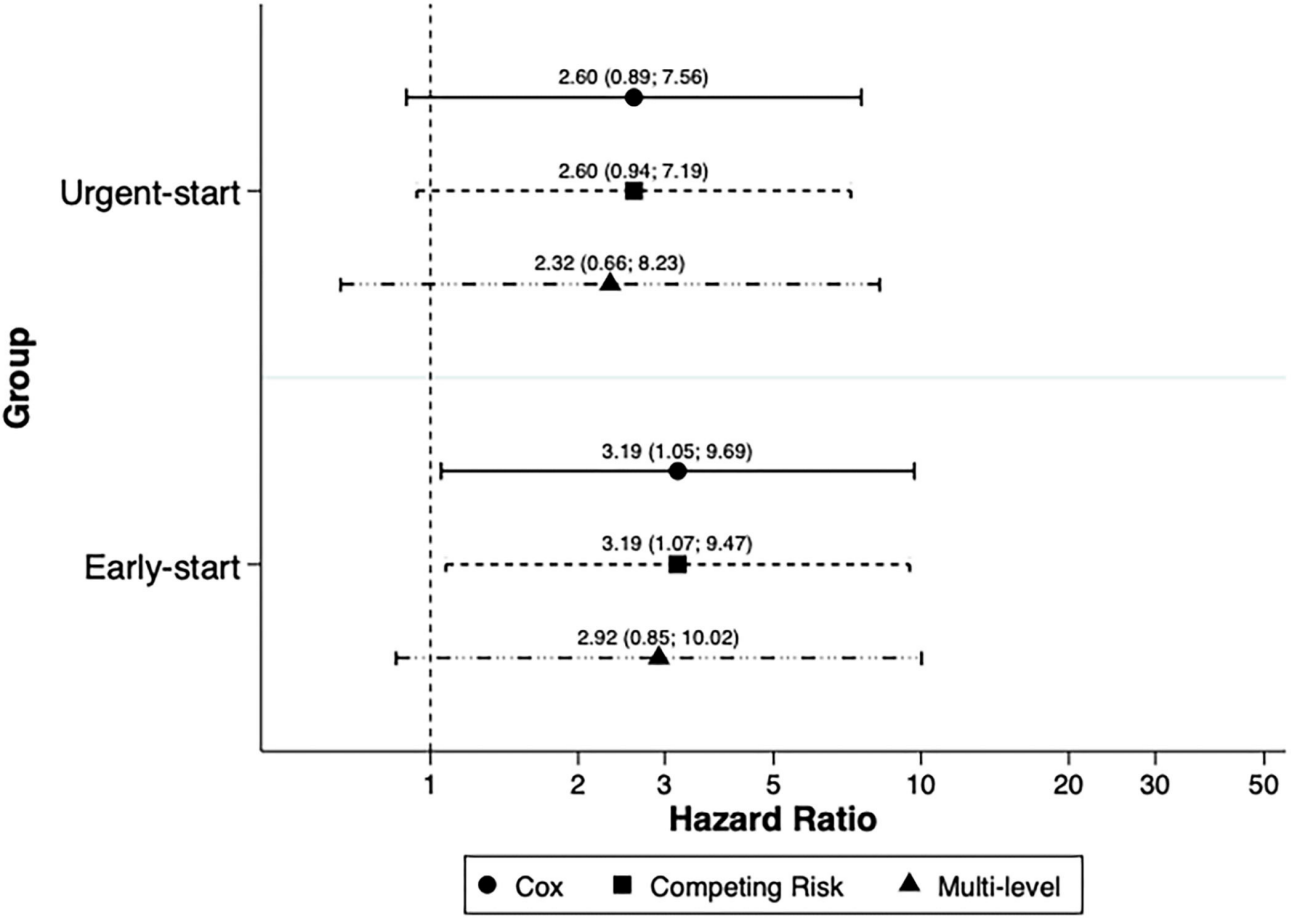
Patients' survival within the first 90 days of follow-up.

There were 25 composite outcomes within the initial 90 days of follow-up, comprising 12 deaths and 13 transfers to HD, with a follow-up time of 1,393.3 months. The survival curves of the study groups were proportional during the first 90 days and had no statistical difference (log rank 130 = 0.21) (Figure 1).

The overall incidence rate of events was 17.9 per 1000 patients/month (95% CI 12.1–26.5). The crude incidence rate of events (per 1000 patients/month) was 23.4 (95% CI 13.0–42.3) for the urgent-start group, 21.9 (95% CI 11.4-42.2) for the early-start group, and 9.7 (4.0–23.4) for the conventional-start group. The causes of death and technique failure within the groups are described in Table 2. Cardiovascular death was the main cause of death in all groups, and refractory peritonitis was responsible for technique failure in four patients from the urgent-start subgroup, but none in the other two groups.

**Table 2:** Events and causes per group within 90 days of dialysis therapy^*^.

	**Urgent-**	**Early-**	**Conventional-**
	**start**	**start**	**start**
**Causes of death**	% (*n* = 5)	% (*n* = 5)	% (*n* = 2)
Cardiovascular	40 (2)	40 (2)	50 (1)
Pulmonary edema	20 (1)	–	–
Peritonitis	20 (1)	20 (1)	–
Sepsis not related to PD	–	40 (2)	50 (1)
Other causes	20 (1)	–	–
**Causes of technique failure**	% (*n* = 6)	(*n* = 4)	(*n* = 3)
Ultrafiltration failure	–	50 (2)	67 (2)
Peritonitis	67 (4)	–	–
Others	–	25 (1)	–
Catheter dysfunction	33 (2)	25 (1)	33 (1)
Exit-site infection	–	–	–

^*^The time at risk for the three groups was 469.5, 410.0, and 513.8 months, respectively.

**Figure 2:**
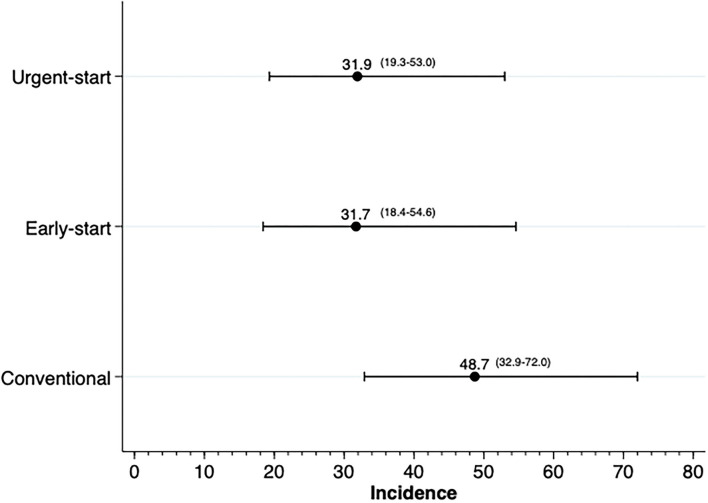
Risk of a composite outcome of death and technique survival.

The best-fit model for the composite outcome was composed of the variable of interest, the presence of residual renal function, history of pre-dialysis care, and anemia. Patients with RRF had a 74% risk reduction for the event (HR 0.24; 95% CI 0.11–0.52), while patients who had seen a nephrologist for more than 3 months prior to dialysis had a 57% risk reduction in comparison with patients without pre-dialysis care (HR 0.43; 95% CI 0.18–1.01). Patients with anemia had an increased risk of the composite event (HR 3.30; 95% CI 1.40–7.80). Figure 2 summarizes the risk of the composite event in the early- and urgent-start groups using three distinct survival models, namely, Cox, competing risk, and a multilevel analysis.

### Laboratory data

Laboratory data were similar between groups at baseline, and the pattern of metabolic control after 3 months also followed a similar behavior. On average, about 40% were anemic at baseline, and this number reduced by almost half after 3 months. The pattern was the same for all subgroups. A similar behavior occurred for hyperkalemia and hyperphosphatemia. Hyperkalemia at baseline was 18% and it was reduced to 8% in 3 months, while for hyperphosphatemia the prevalence diminished from 36 to 20% (Supplementary Figure 2).

### PD-related infections

**Figure 3:**
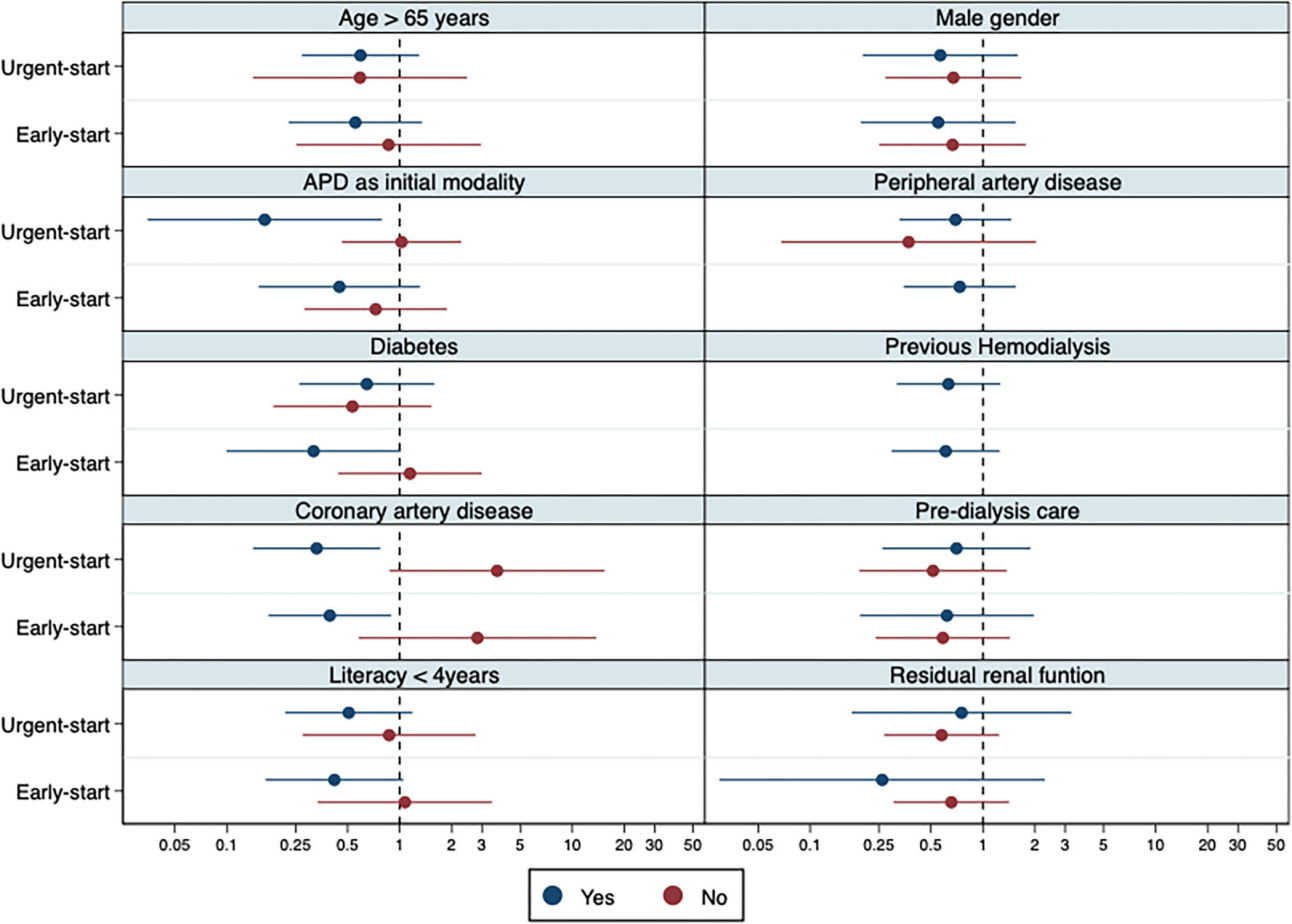
Incidence of any PD-related infection within the initial 90 days of therapy. Legend: The incidence is reported in episodes of PD-related infection (peritonitis and exit-site infection) per 1,000 patient-months.

**Figure 4:**
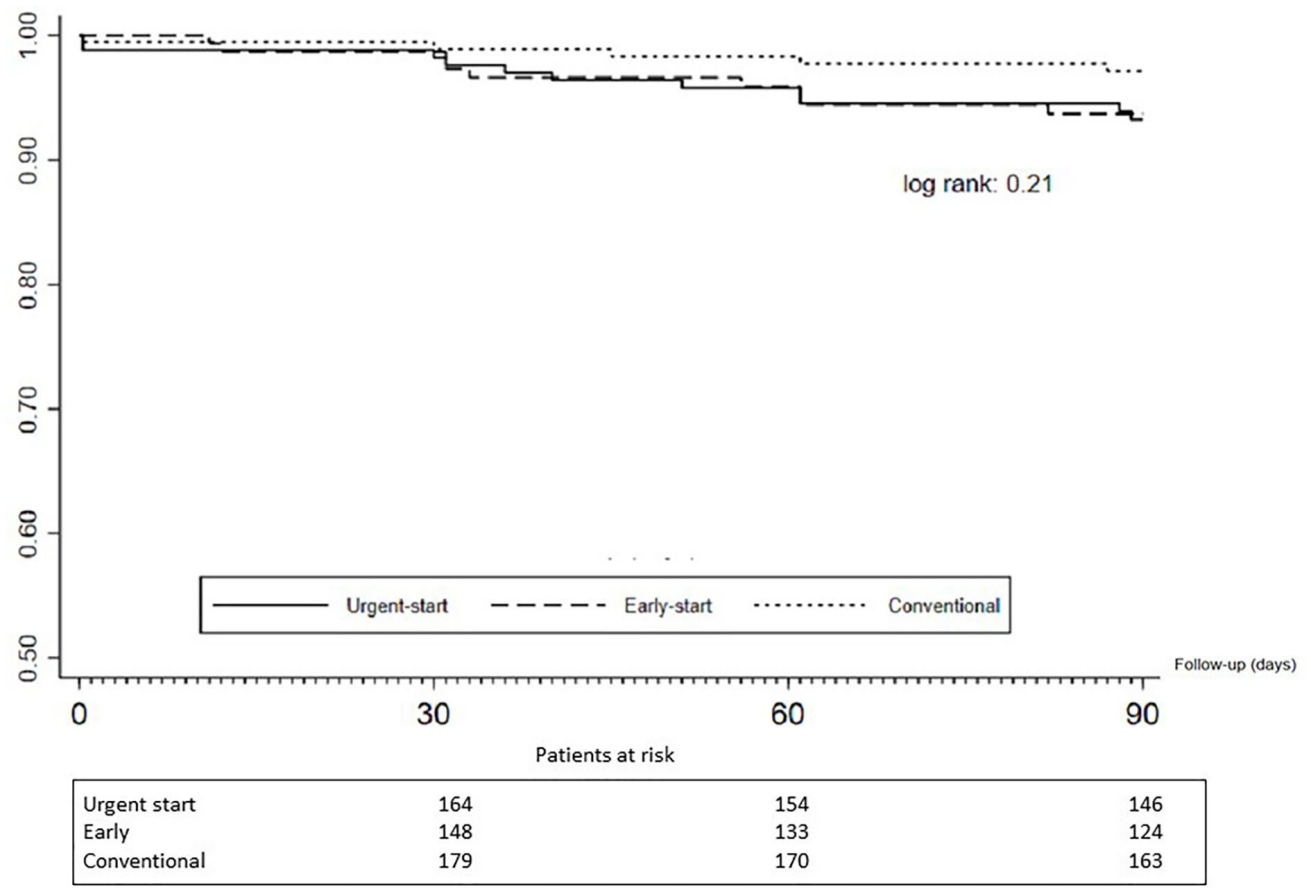
Sub-group interactions: odds ratio for any PD-related infection within 90 days. Legend: The blue lines represent the subgroup with the condition/status on the subtitle and red lines patients without the condition. Given the small number of events in some subgroups, as of peripheral artery disease and previous hemodialysis, some estimators may be missing.

Regarding PD-related infections, there were 53 events in 53 patients, distributed as follows: 8.8% (*n* = 15), 8.5% (*n* = 13), and 13.5% (*n* = 25) for the urgent-, early-, and conventional-start groups, respectively. There was no increased risk of any PD-related infections between groups in comparison with the conventional-start group (urgent-start group: OR 0.62; 95% CI 0.31–1.22; early-start group: OR 0.60; 95% CI 0.29–1.21). The events incidence rate is presented in Figure 3. We also tested for interactions for several potential confounders between early- and urgent-start groups, and the results were homogeneous (Figure 4).

## Discussion

In one of the largest studies derived from a national PD cohort, we reported that urgent-start PD is not associated with lower short-term patient and technique survival with different metabolic controls in comparison with early-start and conventional-start PD.

Unplanned dialysis is common and has been used for a long time in patients with ESKD. Typically, these patients have worse outcomes and were either late referred to a nephrologist or had serious or potentially fatal complications, which requires immediate dialysis (1–3, 8). In this setting, several studies associate this situation with poor clinical results, which have been largely attributed to the use of temporary hemodialysis catheters and to an inadequate metabolic control preceding the initiation of chronic dialysis (14).

In Brazil, this situation is quite common and the demand for unplanned dialysis start is high. There are no reliable data in the country to measure the incidence of unplanned dialysis initiation in patients with ESKD. The number is probably heterogeneous because of its continental dimension and the remarkable social and cultural differences across regions. However, in the capital of the wealthiest state in the country, the need for unplanned dialysis was reported to be around 60% for more than two decades (15). The situation has deteriorated over the past 25 years for reasons linked to the lack of policies to tackle financial issues faced by dialysis centers and the inability to predict the increase in the number of patients to come. The number of facilities has remained stable over decades, while the incidence and prevalence of patients with ESKD rose by 36.7% and 44.7, respectively, from 2008 to 2018, and the proportion of patients using CVC increased from 15.4%, in 2013, to 22.6%, in 2017 (16, 17). The demand exceeded the existing HD capacity, and unplanned PD proved to be a good solution. In our study, 63% of patients who started PD did so by using the PD catheter within the first 2 weeks after catheter implantation. It is important to note, however, that we cannot consider all of them to be unplanned dialysis patients as some individuals from the early-start group may be elective patients depending on the clinic (12). For this reason, a robust multilevel approach is important to diminish the impact of such issues.

The dropout at 90 days in our population was 7.4%, and urgent-start PD was not associated with a higher risk of the composite event. Death was two times as common as technique failure, and the specific causes of events were similar among the three groups (Table 2). Figure 1 depicts the Kaplan–Meier curve, and there is no difference between groups. Long-term (>90 days) results did not change in comparison with the results reported for short-term outcomes (see Supplementary material). These findings reinforce the results of our predecessors that unplanned PD dialysis is not associated with more adverse events, namely, mortality and technique failure (9, 10, 18–23). It is important, in contrast, to emphasize that the total number of events was not large, and the results should be carefully interpreted, due to the potential for the study to be underpowered

Infection is one of the main concerns related to urgent-start PD. For example, the careful choice of the exit-site location in these patients may not be done with the care recommended by the ISPD to facilitate its future cleaning and reduce the chance of inadvertent trauma by the belt (24).

Moreover, the recommended period of keeping the incision dressed for 3–5 days to allow epithelization and healing is not adhered to. However, Pai et al. in a retrospective study of 149 patients found no increased risk for those who used the catheter, although they divided the population using the 2-week threshold (19). Also, in a small single-center study, Nayak et al. demonstrated good results in terms of catheter dysfunction and peritonitis rates, but in only 56 patients (20). Our study found similar results to these two studies. The risk of PD-related infection was similar between the urgent- and early-start groups and even lower when compared with patients from the conventional-start group.

Finally, as late referred patients do not have previous adequate education from the nephrology team, metabolic disturbances may be more common in these individuals. In our study, we noted that the prevalence of anemia, hyperkalemia, and uncontrolled hypertension was similar between groups. In contrast, patients in the urgent-start group had a higher prevalence of hyperphosphatemia compared with those in the other two groups (Supplementary Figure 2). Nevertheless, after 90 days, all groups achieved similar levels of metabolic control: The prevalence of anemia and hyperphosphatemia dropped by almost 50%, reaching on average 20% of the population, while that of hyperkalemia dropped from 50 to 75% to values between 5 and 11%. At 90 days, there was no significant difference in the pattern of patient with systolic blood pressure above 140 mmHg or diastolic blood pressure above 90 mmHg.

Our study has limitations related to its observational design and consequent impossibility of inferring cause and effect. We do not have details about the indication for dialysis initiation in the early-start group because some centers may routinely start PD within this period; the database did not capture whether some individuals required a few sessions of HD; we lack information about the structure of the PD centers; and finally, our analysis could be underpowered, given the small number of clinical events and the brief follow-up times for the primary analysis.

Nevertheless, our study is the only one to our knowledge that adjusts the results using multivariate and multilevel approaches.

## Conclusions

Unplanned PD seems to be a safe and feasible option for treatment for patients with non-dialysis ESKD in urgent need of dialysis. Our results are in line with those of the current literature suggesting that the results of unplanned PD may contribute to reducing the burden over the higher demand for dialysis in some countries, reduce costs for being more financially attractive, and eventually improve the penetration of PD. Finally, randomized controlled trials are needed to confirm these results.

## Data availability statement

All relevant data are within the manuscript to draw results and conclusions. Raw data on cohort including patients exclude from this analysis are available upon reasonable written requests to the institutions Pontifícia Universidade Católica do Paraná, School of Medicine, Rua Imaculada Conceição 1155, Curitiba, PR, 80215-182, Brazil. Requests can also be sent to the author TM (email: thyago.moraes@pucpr.br).

## Ethics statement

The studies involving human participants were reviewed and approved by Ethical Committee of the Pontificia Universidade Católica do Paraná (PUC-PR), register number 448. The patients/participants provided their written informed consent to participate in this study.

## Author contributions

KH wrote the first version of the manuscript. PGB, PB, and RP-F contributed to the study design and revised the article critically for important intellectual content. TM contributed to the concept and design of the work, statistical analysis, and interpretation of data. All authors discussed the results and contributed to the final version of the manuscript.

## Funding

The BRAZPD cohort was funded by Baxter Healthcare, Brazil. The current data extraction and analysis was supported by an investigator-driven study grant provided to the Pontificia Universidade Católica do Paranáa, as part of the Clinical Evidence Council Program from Baxter Healthcare.

## Conflict of interest

Author RP-F received research grants, consulting fees, and speaker honorarium from Baxter Healthcare. Authors PB and TM received consulting fees and speaker honorarium from Baxter Healthcare. Author PB receive occasional speaking honoraria from Baxter Global. The remaining authors declare that the research was conducted in the absence of any commercial or financial relationships that could be construed as a potential conflict of interest.

## Publisher's note

All claims expressed in this article are solely those of the authors and do not necessarily represent those of their affiliated organizations, or those of the publisher, the editors and the reviewers. Any product that may be evaluated in this article, or claim that may be made by its manufacturer, is not guaranteed or endorsed by the publisher.
