# Supplementary figures and images for: Impact of unplanned peritoneal dialysis start on patients' outcomes—A multicenter cohort study
Source: Front Med (Lausanne). 2022 Nov 23;9:717385. doi: 10.3389/fmed.2022.717385 (PMC9727097; doi:10.3389/fmed.2022.717385)

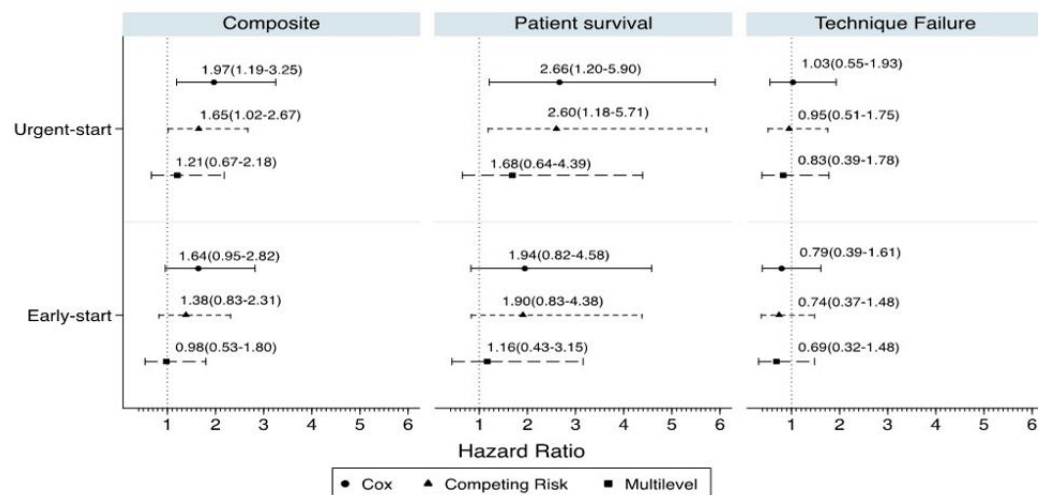

**Figure S1.** Long-term (> 90 days) outcomes of the groups urgent and early peritoneal dialysis start.

Supplement: Supplementary file 1 [file Image_1.pdf]

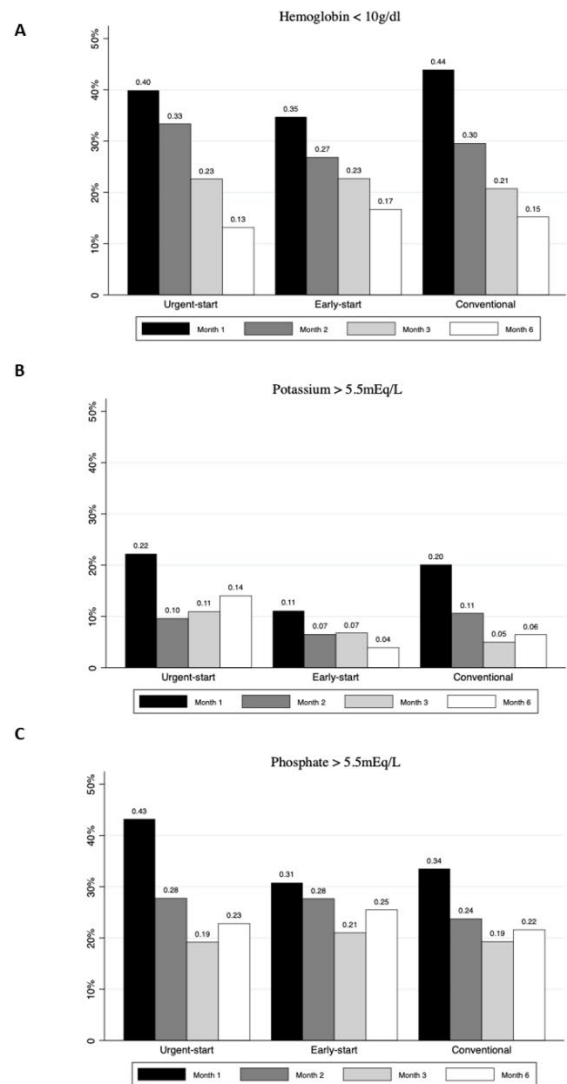

**Figure S2.** Laboratory data over time stratified by group

Supplement: Supplementary file 2 [file Image_2.pdf]
